# Supplementary material for: Functional iron deficiency and outcomes in patients with kidney disease
Source: PLoS One. 2026 Mar 24;21(3):e0343724. doi: 10.1371/journal.pone.0343724 (PMC13012524; doi:10.1371/journal.pone.0343724)
Supplement: Attachment — Submitted filename: Response to reviewers.docx [file pone.0343724.s002.docx]

**PONE-D-25-50596**

**Functional iron deficiency and outcomes in patients with kidney disease**

**Response to reviewers**

**January 2025**

Thank you for your time in reviewing this manuscript and your constructive comments to improve this work. We have responded point-by-point to your feedback and with accompanying tracked changes in the article. We have endeavoured to improve the work based on your suggestions and feedback. We hope that you will favourably consider this edited version for publication.

***Reviewer #1: Cohorts and definitions: The date of the first iron infusion was used as the study start date. Were patients excluded that did not receive iron? This leads onto the question whether iron supplementation was the actual culprit in the cohort of FID?***

Dialysis patients were included in the study from their date of first dialysis. Non-dialysis CKD (ND-CKD) patients were included from their first iron infusion and therefore only CKD patients who had ever received an iron infusion were included. We have added reference to this point in the limitations.

Lines 307-309: “The CKD population was limited to patients who had ever received intravenous iron therapy; therefore, the findings may be subject to selection bias and the population, especially the NID subgroup, is not fully representative of all CKD patients.”

(NID here = non iron deficient patients)

***Results, HD, Cohort: Also, patients in the FID group required higher iron … I suggest adding a definition of what the patients required, i.e.: “ … required higher iron dosages/supplementation…“***

As suggested, we have added “dosages”. The amount required is included in brackets in the sentence.

Lines 141-143: “Also, patients in the FID group required higher iron dosages (FID: 150 vs AID: 86 vs NID:116 mg per month; p=0.048)…”

***CKD cohort: In the CKD cohort patients with FID were significantly older (FID: 72 vs AID: 71 vs NID: 68 years; p=0.001), and a lower proportion were males (FID: 49% vs AID: 38.6% vs NID: 57%; p<0.001). Also …I suggest adding that the comparison relates to the NID cohort.***

Thank you, we have done that.

Lines 198-200: “In the CKD cohort patients with FID were significantly older than those with NID (FID: 72 vs AID: 71 vs NID: 68 years; p=0.001), and a lower proportion were males (FID: 49% vs AID: 38.6% vs NID: 57%; p<0.001).”

***Discussion: “Patients with FID in the HD cohort had a higher iron and ESA requirement, in keeping with the previously reported association of FID and ESA-hyporesponsiveness”. This phrasing/description that higher iron and ESA requirements were higher in the FID cohort contrasts with the lower iron and ESA dosages shown in Table 1 in the AID group. One would expect absolute iron deficiency to lead to physicians responding with an increase in iron dosage to replete iron storage versus NID patients. One could argue that the inappropriate response was possibly the cause for iron deficiency. However, the authors describe in the statistics section that time averaging was used. Thus, I would appreciate clarification.***

Thank you for this point. Time averaging was used. We have added the point about potential inadequate dosing in the AID group to the discussion. However, it is well recognised that haemoglobin response to iron (and ESA) in patients with AID is far better than in FID which will partly account for the lower dosing seen in AID

Lines 273-276: “Patients with FID in the HD cohort had a higher iron and ESA requirement, in keeping with the previously reported association of FID and ESA-hyporesponsiveness (16, 17). Patients with FID had the highest iron dosages, followed by those with NID and then AID.”

***Reviewer #2: The retrospective study presented in the current manuscript has several strengths, including hard clinical outcomes, a well-designed and detailed methodology with carefully chosen selection criteria, a large sample size, and approximately three years of follow-up. These strengths help counterbalance the limitations inherent to its retrospective design.
I have only minor suggestions:
- Since functional iron deficiency is clearly associated with systemic inflammation, the discussion and interpretation of the results should place stronger emphasis on the fact that functional iron deficiency is very likely a marker of systemic inflammation, and that the observed impact on outcomes can be explained by the effects of inflammation.***

Thank you for your review and suggestion. As you have suggested, we have edited the discussion to emphasise the role of inflammation.

Lines 290-301: “C-reactive protein was higher in those patients with FID in both cohorts and a manifestation of systemic inflammation. Hepcidin, a hormone released by the liver in systemic inflammation, has a major effect upon iron physiology by promoting degradation and internalisation of the main cellular iron exporter protein, ferroportin, which results in iron being ‘locked in’ cells (especially macrophages and hepatocytes, but also in gut enterocytes) and unavailable for transport to tissues requiring iron such as the bone marrow. Indeed, ferroportin, the hepcidin target is present in other tissues including the renal tubules, cardiac myocytes, vascular smooth muscle cells, lung epithelia and pancreatic beta cells (23-26). Iron sequestration within these tissues has already been proposed to contributing to poor outcomes, including poor response to intravenous iron therapy in the context of FID (27). Systemic inflammation is well recognized as being associated with poor outcomes and the poorer outcomes in the FID subgroups may, partly because of the above disordered physiology, be related to the association of FID with chronic systemic inflammation.”
